# 6-Gingerol Ameliorates Adiposity and Inflammation in Adipose Tissue in High Fat Diet-Induced Obese Mice: Association with Regulating of Adipokines

**DOI:** 10.3390/nu15153457

**Published:** 2023-08-04

**Authors:** Kyung Hee Hong, Min Young Um, Jiyun Ahn, Tae Youl Ha

**Affiliations:** 1 Department of Food Science and Nutrition, Dongseo University, Busan 47011, Republic of Korea; hkhee@gdsu.dongseo.ac.kr; 2 Division of Food Functionality Research, Korea Food Research Institute, Wanju-gun 55365, Republic of Korea; myum@kfri.re.kr (M.Y.U.); jyan@kfri.re.kr (J.A.); 3 Department of Food Biotechnology, University of Science & Technology, Daejeon 34113, Republic of Korea

**Keywords:** 6-gingerol, obesity, adipogenesis, adipokine, inflammation

## Abstract

We investigated the effects of 6-gingerol on adiposity and obesity-induced inflammation by focusing on the regulation of adipogenesis and adipokines in white adipose tissue (WAT) of diet-induced obese mice. C57BL/6 mice were fed a high-fat diet (HFD) containing 0.05% 6-gingerol for 8 weeks. 6-Gingerol supplementation significantly reduced body weight, WAT mass, serum triglyceride, leptin and insulin levels, and HOMA-IR in HFD-fed mice. Additionally, the size of adipocytes in epididymal fat pads was reduced in HFD-fed mice by 6-gingerol supplementation. 6-Gingerol reduced the mRNA and protein levels of adipogenesis-related transcription factors, such as SREBP-1, PPARγ, and C/EBPα in WAT. Furthermore, 6-gingerol suppressed the expression of lipogenesis-related genes, such as fatty acid synthase and CD36 in WAT. Adiponectin expression was significantly increased, whereas inflammatory adipokines (leptin, resistin, TNF-α, MCP-1, and PAI-1) and the macrophage marker F4/80 were significantly reduced in the WAT of HFD-fed mice by 6-gingerol supplementation. In conclusion, 6-gingerol effectively contributed to the alleviation of adiposity and inflammation in WAT, which is associated with the regulation of adipokines in diet-induced obese mice.

## 1. Introduction

Obesity is an excessive accumulation of fat in white adipose tissue (WAT) that leads to metabolic disorders. WAT is an endocrine organ that plays a fundamental role in the regulation of metabolism and homeostasis by secreting several biologically active peptides, collectively known as adipokines. For this reason, increased adiposity leads to chronic inflammation, resulting in increased production of pro-inflammatory adipokines, such as TNF-α, MCP-1, IL-6, and PAI-1, and decreased production of anti-inflammatory adipokines, such as adiponectin. Furthermore, the number of macrophages in WAT increases with obesity. Macrophages are responsible for most of the cytokine production, and the dysregulation of adipokine secretory patterns has been identified as a link between obesity and obesity-related disorders [1,2,3]. One potential strategy for reducing obesity-related inflammation is the consumption of food components reported to have anti-inflammatory effects.

Ginger (rhizome of *Zingiber officinale*) is widely used as a spice and herbal medicine. The major chemical components of ginger rhizomes are gingerols, shogaols, and zingerones. Among these components, 6-gingerol is the main pungent component and is believed to exert a variety of remarkable pharmacological and physiological activities, including anti-inflammatory [4,5,6,7], anti-diabetic [8,9], anti-hepatic steatosis [10], anti-oxidant [6,11], and anti-cancer [12,13] effects. 6-Gingerols have also been reported to have anti-obesity properties, in 3T3-L1 preadipocytes, 6-gingerol shows inhibitory adipocyte differentiation [14,15,16]. In addition, animal studies have shown that 6-gingerol reduces body fat accumulation and the extent of lipogenesis in the liver [17,18] and improves insulin resistance [18,19,20,21] and serum lipid profile [18,20,21,22] in obese rodents. Furthermore, 6-gingerol reduced adipogenesis, along with inflammatory cytokine expression in 3T3-L1 cells [23]. In obese rodents, 6-gingerols modulated the expression of inflammatory mediators in the liver [20,21]. Although some researchers have focused on the anti-obesity effects of 6-gingerol in vitro and in vivo, few studies have elucidated its anti-obesity action on the modulation of obesity-associated inflammatory conditions. Furthermore, little is known about the molecular mechanisms underlying the action of 6-gingerol on WAT in diet-induced obesity models.

In this study, we investigated whether supplementation with 6-gingerol in diet-induced obese mice could influence body fat accumulation in the adipose tissue and how this affects obesity-induced inflammatory conditions, focusing on the expression of genes involved in adipogenesis and inflammation in WAT.

## 2. Materials and Methods

### 2.1. Animals and Diets

The protocol of this study was approved by the Institutional Animal Care and Use Committee at the Korea Food Research Institute (KFRI-M-18001). Four-week-old male C57BL/6J mice (OrientBio, Seongnam, Korea) were adapted for 1 week and then randomly assigned to three experimental groups (*n* = 10 per group). Each group was fed different experimental diets and housed for 8 weeks. The experimental diets were a normal diet (ND), a high-fat diet (HFD), and a high-fat diet containing 0.05% 6-gingerol (HFD + 6G). The HFD consisted of 20% fat and 0.5% cholesterol added to the AIN-76A diet so that 45% of energy was provided by fat. The dose of 6-gingerol (Sigma-Aldrich, St. Louis, MO, USA) used in the experimental diet was selected based on previous animal studies [17,24]. The composition of the experimental diets is shown in Supplementary Table S1. Body weight and food intake were assessed three times a week during the 8-week experimental period. Water and food were provided ad libitum. Animals were housed in individual cages and maintained at room temperature of 24 ± 1 °C and humidity of 55 ± 5% with a 12 h light/dark cycle.

After 8 weeks, the animals were sacrificed after 12 h of fasting. Blood was collected from the abdominal aorta using heparin tube and centrifuged at 1500× *g* for 20 min, and serum was stored at −80 °C for further analyses (lipids, glucose, insulin, leptin, and adiponectin). Epididymis and peripheral adipose tissue were obtained by excision, weighed, snap-frozen in liquid nitrogen, and stored at −80 °C for analyses.

### 2.2. Biochemical Analysis

Serum triglyceride (TG), free fatty acids (FFA), total cholesterol (TC), HDL-cholesterol, and glucose levels were measured by commercial kits. Insulin, leptin, and adiponectin levels were analyzed by ELISA kits. Analyses were performed according to the manufacturer’s assay instructions. The product codes of the kits are listed in Supplementary Table S2. Insulin resistance was estimated by the homeostasis model assessment of insulin resistance (HOMA-IR), calculated from glucose and insulin levels in serum [25].

### 2.3. Histological Analysis

To measure adipocytes in WAT, the isolated epididymal WAT was fixed in 10% neutral formalin solution and paraffin-embedded specimens were prepared. Specimens were cut into 5-μm thick sections and stained with hematoxylin and eosin (H&E) to observe the size of adipocytes under a light microscopy (Olympus BX50, Tokyo, Japan), and digital images were recorded. The mean diameter size of adipocytes in each experimental group was quantified using ImageJ software (version 1.53e; NIH, Bethesda, MD, USA).

### 2.4. Western Blot Analysis

The isolated epididymal WAT was homogenized in lysis buffer using a FastPrep-24 sample preparation system (MP Biomedicals LLC, Solon, OH, USA), centrifuged, and the supernatant collected for protein extraction. The total protein concentration was estimated using Bradford assay. The method for measuring protein expression was as follows. Equal amounts of protein were electrophoresed using an 8–10% SDS-polyacrylamide gel, followed by protein transfer to a polyvinylidene fluoride (PVDF) membrane. After transfer, the membrane was blocked with TTBS (0.05% Tween-20 in Tris-buffered saline) solution containing 5% non-fat dry milk and incubated with primary antibodies for 4 h. The primary antibodies used were as follows C/EBPα, PPARγ, SREBP-1, FAS, and CD36 (Cell Signaling Technology, Beverly, MA, USA). Secondary antibodies (peroxidase-conjugated IgG) were added to bind to the primary antibodies, and protein expression levels were detected using an ECL detection system. Protein expression levels were also calculated using ImageJ software.

### 2.5. Geme Expression Analysis by Quantitative Real-Time RT-PCR

Total RNA was extracted from epididymal WAT using an RNase kit (Intron, Seongnam, Republic of Korea), and the concentration of each RNA sample was quantified on a Nanodrop 2000C spectrophotometer (Thermo Fisher Scientific, Waltham, MA, USA) using UV absorbance. Then, 1 μg of RNA was used to reverse transcribe to cDNA (Maxime RT-PCR premix kit, TOYOBO, Osaka, Japan). Primer sequences required for gene expression analysis are shown in Supplementary Table S3. The mRNA expression levels of target genes were measured by amplification by quantitative real-time PCR (qPCR), using SYBR^®^ Green PCR Master Mix (TOYOBO, Osaka, Japan) on a StepOnePlus Real-Time PCR detection system (Applied Biosystems, Waltham, MA, USA). Relative quantification of mRNA expression was performed by using 2^−ΔΔCT^ method [26].

### 2.6. Statistical Analysis

The results of each group were expressed as mean ± standard error (SE). SPSS statistical software (version 14.0; SPSS Inc., Chicago, IL, USA) was used, we analyzed the significance of the mean difference between groups using ANOVA, and verified the mean difference by performing Tukey’s multiple range test. Significance levels for all statistical analyzes were set at *p* < 0.05.

## 3. Results

### 3.1. 6-Gingerol Reduces Body Weight Gain and Adipose Hypertrophy

**Figure 1:**
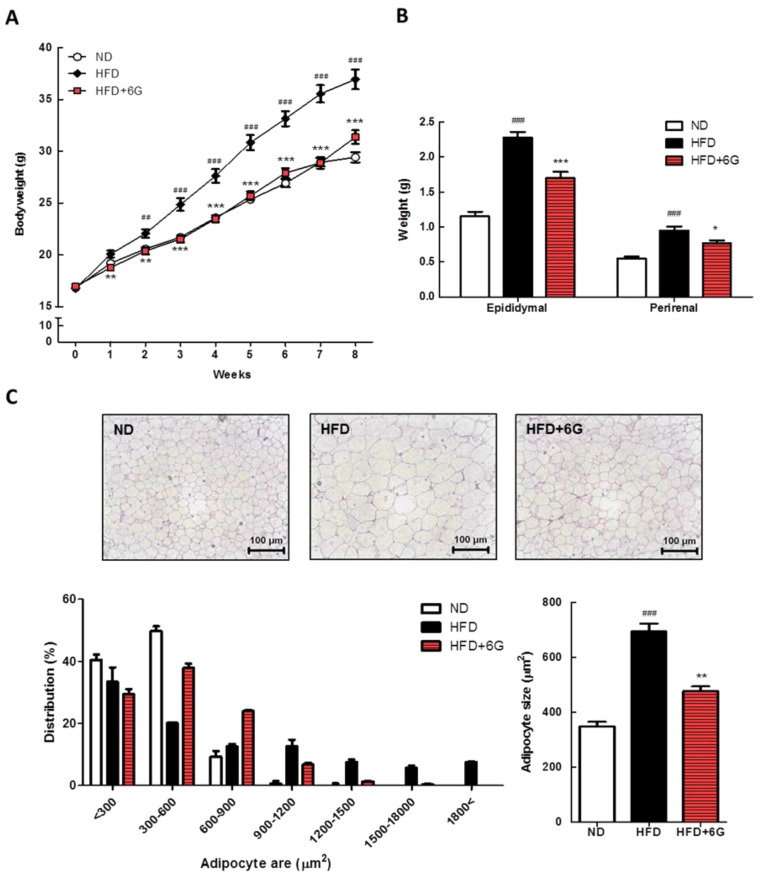
Body weight (**A**), WAT weight (**B**), and epididymal WAT morphology and distribution of adipocyte size (**C**) in 6-gingerol supplemented HFD-induced obese mice. Representative photographs of epididymal WAT stained with H&E, shown at ×200 magnification. The adipocyte size from three different groups was determined using ImageJ software (NIH). The frequency distribution of adipocyte surface in epididymal WAT. Values are means ± SE. ^##^ *p* < 0.01; ^###^ *p* < 0.001 versus ND group. * *p* < 0.05; ** *p* < 0.01; *** *p* < 0.001 versus HFD group.

To determine whether 6-gingerol affects body weight, we measured the body weight of mice for 8 weeks. The body weights of HFD-fed mice increased significantly higher than those of ND-fed mice. In contrast, the HFD + 6G group showed a 28.8% decrease in body weight gain compared to the HFD group (Figure 1A); however, no significant differences in food intake were observed between the experimental groups (Table 1). Similarly, the weights of epididymal and perirenal WAT increased by 198.3% and 172.7%, respectively, in the HFD group; however, this increase was attenuated by 6-gingerol supplementation (*p* < 0.001 and *p* < 0.05, respectively; Figure 1B). The effect of 6-gingerol on the WAT weight was determined by histological examination. As shown in Figure 1C, the size of epididymal adipocytes in the HFD group was greater than that in the ND group, whereas 6-gingerol supplementation effectively reduced the adipocyte size (*p* < 0.01). These results indicate that 6-gingerol suppressed HFD-induced body weight gain, WAT mass increase, and adipocyte hypertrophy.

### 3.2. 6-Gingerol Reduces Serum Lipids, Insulin, and Leptin Levels and Improves Insulin Resistance

Next, the effects of 6-gingerol on serum lipids, glucose, insulin, leptin, and adiponectin levels and HOMA-IR were evaluated. As shown in Table 1, serum TG levels were not significantly different between ND group and HFD groups, but were significantly lower in the HFD + 6G group. Consistent with this result, despite being statistically insignificant, serum FFA levels also were lower in the HFD + 6G group than in the HFD group. Mice fed a HFD had significantly higher TC and HDL-C levels than those in the ND group, but there was no significant effect of 6-gingerol. Compared to the HFD group, the serum levels of insulin and leptin were reduced by 33.1% and 43.7%, respectively, following 6-gingerol supplementation. Fasting glucose levels were significantly increased in HFD group and were slightly decreased by the supplementation of 6-gingerol, although this was not statistically significant. In addition, the insulin resistance index HOMA-IR was significantly higher in the HFD group than in the ND group by approximately 2-fold, whereas it was decreased by 45.6% in the HFD + 6G group. Finally, the serum adiponectin levels in the HFD group were lower than those in the ND group, whereas the HFD + 6G group had significantly higher levels.

### 3.3. 6-Gingerol Modulates the Adipogenesis Involved Gene Expression in WAT

**Figure 2:**
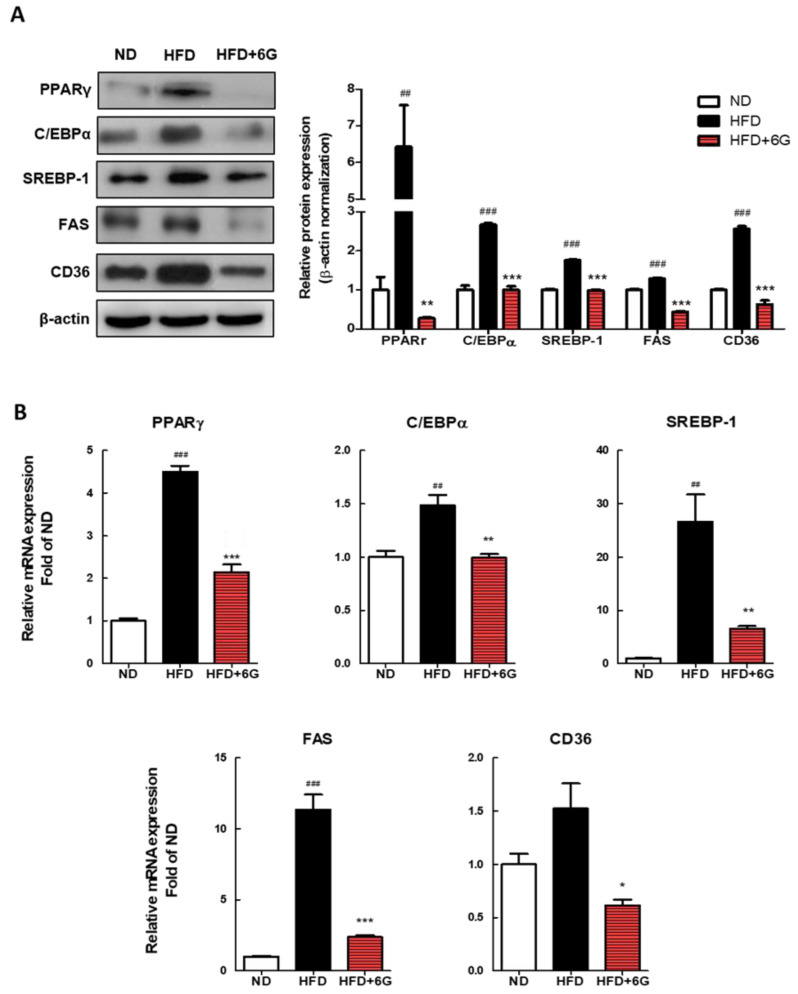
Effect of 6-gingerol on the expression of protein (**A**) and mRNA (**B**) involved in adipogenesis and lipogenesis in the epididymal WAT of HFD-induced obese mice. Protein levels were determined by Western blot analysis and normalized to β-actin. The mRNA levels of target genes were determined by real-time qRT-PCR and normalized to β-actin. Values are means ± SE. ^##^ *p* < 0.01; ^###^ *p* < 0.001 versus ND group. * *p* < 0.05; ** *p* < 0.01; *** *p* < 0.001 versus HFD group.

To elucidate the mechanism by which 6-gingerol reduces WAT mass and adipocyte size, we measured the expression of adipogenesis-related proteins and mRNA in WAT using Western blotting and qRT-PCR. As shown in Figure 2A, in the HFD + 6 group, the mRNA levels of adipocyte differentiation-associated genes, including adipogenic transcription factors, such as C/EBPα, PPARγ, and SREBP-1, exhibited significant reductions of 33.1%, 56.9%, and 75.4%, respectively, compared to the HFD group. Expression levels of the FAS and CD36, which are target genes of these transcription factors, also were lower in the 6-gingerol supplemented group (−79.1% and −59.2%, respectively). Immunoblotting results were consistent with the mRNA data, showing the downregulation of adipogenic genes by 6-gingerol supplementation in epididymal WAT (Figure 2B). These results suggest that 6-gingerol suppresses adipogenesis and lipogenesis, and that 6-gingerol induces a reduction in adiposity resulting from the inhibition of fat accumulation in adipocytes and from failed differentiation into mature adipocytes during HFD feeding.

### 3.4. 6-Gingerol Modulates the Inflammation Involved Gene Expression in WAT

**Figure 3:**
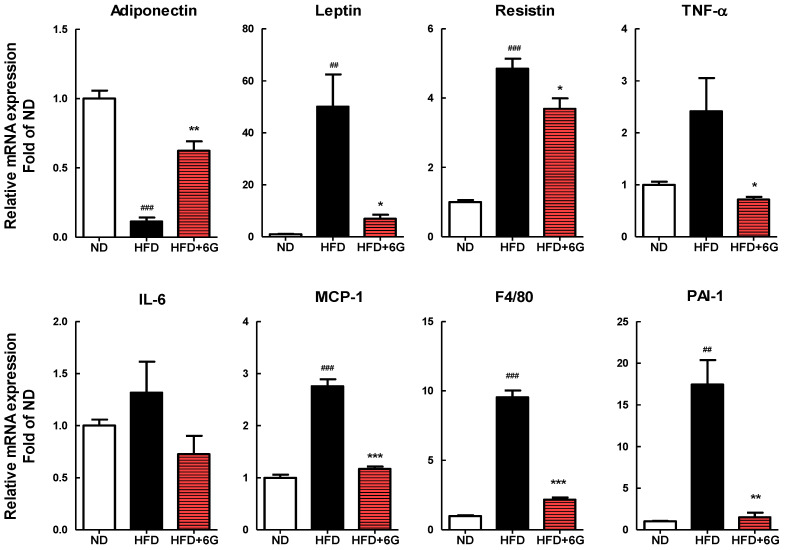
Effect of 6-gingerol on the expression of mRNA involved in inflammation in the epididymal WAT of HFD-induced obese mice. The mRNA levels of target genes were determined by real-time qRT-PCR and normalized to β-actin. Values are means ± SE. ^##^ *p* < 0.01; ^###^ *p* < 0.001 versus ND group. * *p* < 0.05; ** *p* < 0.01; *** *p* < 0.001 versus HFD group.

Based on the active role of adipokines in obesity-related inflammation, we assessed the effects of 6-gingerol on adipokine expression in epididymal WAT. As shown in Figure 3, supplementation with 6-gingerol resulted in a significantly lower leptin, resistin, TNF-α, MCP-1, and PAI-1 mRNA levels in epididymal WAT by 86.2%, 24.0%, 70.3%, 57.6%, and 91.4%, respectively, compared to that in the HFD group. In addition, the mRNA expression of the macrophage marker F4/80 was 77.2% lower in the HFD + 6G group compared in the HFD group. Adiponectin mRNA expression was significantly higher in the HFD + 6G group compared with that in the HFD group (5.5-fold increase; *p* < 0.05). Thus, supplementation with 6-gingerol not only inhibited macrophage infiltration and the mRNA expression of inflammatory adipokines, but also enhanced the mRNA expression of adiponectin in WAT.

## 4. Discussion

We investigated the protective effects of 6-gingerol against HFD-induced obesity in mice. Our study demonstrated that 6-gingerol supplementation successfully reduced body weight gain and fat accumulation in WAT. Typically, reducing food intake results in a reduction in body fat; however, we discovered that the experimental groups had similar food intakes. The epididymal and perirenal WAT weights were lower in the HFD + 6G group compared with those in the HFD group, which was accompanied by a decrease in adipocyte size. Additionally, the analysis of adipocyte distribution in epididymal WAT showed a change in distribution toward smaller adipocytes in the HFD + 6G group. Consistent with our findings, in previous reports, 6-gingerol supplementation effectively attenuated the increase in WAT mass [17,19,22], as well as adipocyte size [21], in rodents with HFD-induced obesity. These demonstrate that 6-gingerol could inhibit HFD-induced adipocyte hypertrophy in WAT, thereby effectively suppressing body fat accumulation and body weight gain.

To understand the potential mechanisms responsible for the anti-obesity action of 6-gingerol, we investigated the expression of genes related to adipogenesis and lipogenesis in WAT. PPARγ and C/EBPα are transcription factors that play a key role in adipogenesis [27]. They regulate several adipocyte-encoding proteins and enzymes involved in adipocyte differentiation, lipogenesis, and insulin sensitivity [28,29,30,31]. SREBP-1 is an important lipogenic transcription factor that plays a critical role in lipid metabolism in WAT [32]. SREBP-1 is also involved in adipogenesis by stimulating PPARγ and C/EBPα [30]. Specifically, PPARγ is activated by SREBP-1, which induces the expression of lipogenic genes, such as FAS and CD36 [28,33]. FAS is a major lipogenic enzyme, catalyzing the reaction that biosynthesizes fatty acids from the two-carbon molecule acetyl-CoA. CD36, a fatty acid importer, is expressed in WAT and promotes fatty acid uptake into adipocyte. Our results showed that 6-gingerol supplementation significantly suppressed the increase in mRNA and protein expression of SREBP-1, PPARγ, and C/EBPα induced by HFD feeding in WAT. In agreement with these results, the expression of the PPARγ and SREBP-1 targeted lipogenic genes FAS and CD36 was also significantly reduced in WAT following 6-ginerol treatment. In previous in vitro studies, 6-gingerol downregulated the gene expression of PPARγ, C/EBPα, SREBP-1, and FAS [23], accompanied by inhibition of adipogenesis and lipid accumulation in 3T3-L1 adipocytes [14,15]. Furthermore, 6-gingerol treatment downregulated the hepatic expression of SREBP-1, PPARγ, and FAS, which was accompanied by the suppression of body weight gain in HFD-induced obese rats [20]. Cumulatively, these findings demonstrate that 6-gingerol exerts its anti-adipogenic and anti-lipogenic effects by suppressing the synergistic activation of PPARγ, C/EBPα, and SREBP-1, thereby contributing to the amelioration of adiposity in HFD-induced obesity.

Furthermore, our findings indicated that, along with downregulation of these transcription factors, 6-gingerol inhibited the HFD-induced increase in serum TG and FFA. Obesity is recognized as a major risk factor for the development of hyperlipidemia. In obesity, enlarged adipocytes increase the release and delivery of FFAs to the liver, leading to promoted TG biosynthesis [1]. In a study by Saravanan et al. [18], 6-gingerol treatment improved the lipid profile by significantly reducing serum TG, FFA, TC, VLDL, and LDL-C levels, and increasing HDL-C levels in HFD-induced obese rats. Similar to our findings, 6-gingerol suppressed PPARγ and C/EBPα expression in WAT accompanied by a reduction in serum TG in HFD-induced obese mice [21]. Therefore, it is suggested that 6-gingerol reduces the extent of lipogenesis by downregulating lipogenic transcription factors, such as SREBP-1, PPARγ, and C/EBPα, leading to a reduced expression of FAS, which, in turn, attenuates hyperlipidemia associated with adiposity.

Obesity is closely related with triggering chronic low-grade inflammation in the WAT, which contributes significantly to the development of insulin resistance, a key feature of metabolic disorders [2]. In an obese state, the expansion of WAT disrupts the normal secretion pattern of adipokines, leading to an imbalance characterized by an increased production of pro-inflammatory adipokines and a corresponding decrease in anti-inflammatory adipokines. This shift in the balance of adipokine secretion promotes a state of chronic low-grade inflammation within the WAT [1]. Furthermore, it has been established that the inflammatory state associated with obesity arises due to a combination of factors, including augmented macrophage infiltration into WAT and upregulated expression and secretion of pro-inflammatory adipokines [3]. For example, macrophages from HFD-fed obese mice infiltrated into WAT and the expression of pro-inflammatory cytokines, including TNF-α, MCP-1, and IL-6, increased in WAT, whereas plasma adiponectin decreased [34]. In particular, visceral obesity is considered to be strongly associated with fat-related inflammation, with studies measuring adiposity by BMI, waist circumference, and waist-hip ratio showing higher levels of leptin, IL-6, and TNF-α and lower levels of adiponectin, IL-10, and IL-8 in subjects with high adiposity [35].

Recently, research results on food components or phytochemicals that ameliorate obesity-associated inflammation have emerged. Basil seed [36] and rooibos [37] inhibited adipogenesis and pro-inflammatory cytokine expression in vitro, and anthocyanins attenuated obesity and associated inflammation by modulating the secretion of inflammatory adipokines in several animal and clinical studies [38]. Here, we observed that HFD induced the enlargement of adipocytes and significantly increased the mRNA levels of pro-inflammatory adipokines, such as TNF-α, MCP-1, PAI-1, leptin, and resistin in the WAT. By contrast, supplementation with 6-gingerol restored the expression of these proinflammatory adipokine genes. On the contrary, the serum levels and mRNA expression of adiponectin, an anti-inflammatory adipokine, were increased by 6-gingerol supplementation. Furthermore, the expression of the macrophage marker F4/80 was lower in the WAT of mice fed a 6-gingerol containing HFD than in mice fed only an HFD, suggesting a potential immunoregulatory function for 6-gingerol in WAT. Thus, these results suggest that the potential positive effect of 6-gingerol on HFD-induced obesity may be partly attributed to its capacity to alleviate inflammation in WAT, leading to the suppression of pro-inflammatory gene expression and the enhancement of anti-inflammatory gene expression in WAT. Few studies have investigated the effects of 6-gingerol on adipokine gene expression and subsequent inflammation. Choi et al. [23] have shown that 6-gingerol decreases the expression of pro-inflammatory cytokines including TNF-α, MCP-1, IL-1β, and IL-6, and increases the expression of the anti-inflammatory cytokine IL-10 in 3T3-L1 adipocytes. In addition, in HFD-fed rats, 6-gingerol treatment downregulated the expression of TNF-α, resistin, and P65 in WAT [22].

In response to the expansion of fat mass, the release of adipokines from both adipocytes and infiltrating macrophages within WAT initiates a state of low-grade chronic inflammation and contributes to the development of insulin resistance [3,39]. Particularly, TNF-α, a pro-inflammatory cytokine, is secreted in large amounts by WAT, and its elevated levels are believed to play a significant role in the pathogenesis of insulin resistance in obesity [1]. TNF-α upregulates gene expression of many pro-inflammatory adipokines, such as leptin, resistin, IL-6, and PAI-1 [25]. Furthermore, TNF-α blocks insulin action on GLUT4 and lipoprotein lipase and counteracts lipogenic actions of PPARγ and C/EBPα [40]. These actions result in elevated plasma FFA and TG levels, thereby triggering insulin resistance [25,41]. Other pro-inflammatory cytokines secreted by the WAT, such as IL-6, MCP-1, and PAI-1, have been suggested to contribute to the development of insulin resistance in obesity [1,25,42]. In contrast, adiponectin, a crucial anti-inflammatory adipokine, exerts beneficial effects by enhancing insulin sensitivity in both muscle in liver and by reducing serum levels of FFA, TG, and glucose [3]. The anti-inflammatory effect of adiponectin is mediated by inhibition of the TNF-α accompanied by an elevated inflammatory response and suppressed inflammatory cytokine production [3]. Additionally, adiponectin inhibits macrophage chemotaxis and inflammatory events, which are promoted by IL-6 and PAI-1 [25]. Leptin is a hormone that is abundantly expressed in WAT and is essential for the maintenance of energy and glucose homeostasis. Obesity-induced hyperleptinemia triggers the upregulation of pro-inflammatory cytokines while downregulating the anti-inflammatory cytokine [1]. Leptin is a powerful chemoattractant for macrophages [3], and it improves insulin sensitivity in the liver and skeletal muscles but impairs insulin signaling in adipocytes [1,25]. Resistin is also an adipokine secreted to a much greater extent by visceral WAT and has been shown to promote both inflammation and insulin resistance [1,25]. In the obese state, adipocyte hypertrophy increases the production of pro-inflammatory adipokines, leading to adipocyte-derived inflammation, which, in turn, inhibits insulin signaling and eventually leads to insulin resistance.

Therefore, our results suggest that 6-gingerol modulates the gene expression of adipokines in the WAT of HFD-induced obese, thereby potentially contributing to the improvement of insulin sensitivity. This study found that 6-gingerol supplementation reduced serum fasting glucose and insulin levels and improved HOMA-IR scores in HFD-fed obese mice. In previous studies, 6-gingerol has been shown to exert hypoglycemic effects and improve insulin resistance in type 2 diabetic [8,9] and HFD-induced obese rodents [20,21]. While oral glucose tolerance was not directly assessed herein, the potential effects of 6-gingerol were inferred from the findings of a previous study. In a study of type 2 diabetic mice, 6-gingerol supplementation was shown to result in a decrease in the area under the curve (AUC) of the oral glucose tolerance test [9].

Based on this, we speculated that the effect of 6-gingerol supplementation on insulin resistance may, in part, be secondary to the suppression of adipocyte-derived inflammation by modulating adipokine gene expression. Moreover, inflammatory adipokines and macrophage infiltration into WAT are involved in the increase in serum TG levels and the pathogenesis of hyperlipidemia [1]. Thus, it is conceivable that the serum lipid-lowering effects of 6-gingerol observed herein are related to the suppression of obesity-related inflammation.

Our study has certain limitations. Firstly, in this study, the effects of 6-gingerol were investigated at a single dose; consequently, we were unable to assess the extent to which different doses of 6-gingerol may influence the observed outcomes. Secondly, we did not incorporate a positive control group in our experimental design, which could have been used to compare the effects of 6-gingerol. Therefore, future research will be needed to determine the dose-dependent response of 6-gingerol and include a positive control to better understand its anti-obesity and anti-inflammatory effects.

## 5. Conclusions

Our investigation demonstrated that 6-gingeroll effectively ameliorates adiposity and obesity-associated inflammation in WAT. The beneficial effects of 6-gingerol arise from its ability to suppress adipogenesis and lipogenesis by interfering with the expression of transcription factors such as SREBP-1, PPARγ, and C/EBPα. Moreover, 6-gingerol exerts anti-inflammatory effect in WAT by ameliorating the obesity-induced dysregulation of adipokine expression. The findings of this study provide valuable insights into the mechanisms through which 6-gingerol influences obesity-related changes in adipose tissue function, leading to improved adiposity, reduced inflammation, and alleviated insulin resistance. These results suggest that the importance of 6-gingerol as a promising phytochemical with potential implications for combatting obesity and related metabolic disease.

## Figures and Tables

**Table 1:** Food intake and biochemical parameters in serum of mice fed HFD supplemented with 6-gingerol.

	ND	HFD	HFD + 6G
Food intake (g/day)	3.29 ± 0.17	3.19 ± 0.05	3.18 ± 0.07
Serum TG (mg/dL)	50.37 ± 2.00	51.29 ± 1.97	42.16 ± 2.33 *
Serum FFA (μEg/L)	590.23 ± 77.36	543.69 ± 66.70	404.96 ± 60.89
Serum TC (mg/dL)	81.24 ± 22.6	128.43 ± 3.63 ^###^	134.79 ± 5.06
Serum HDL-C (mg/dL)	42.43 ± 2.14	59.30 ± 5.47 ^##^	60.55 ± 2.80
Serum leptin (ng/mL)	28.47 ± 3.07	81.69 ± 8.35 ^###^	46.03 ± 4.70 ***
Serum adiponectin (mg/dL)	13.73 ± 0.05	11.21 ± 0.03 ^#^	14.88 ± 0.08 **
Serum glucose (mg/dL)	274.12 ± 17.73	386.77 ± 42.16 ^#^	315.76 ± 17.87
Serum insulin (pg/mL)	364.11 ± 17.38	520.86 ± 68.07 ^#^	348.67 ± 46.90 *
HOMA-IR	6.18 ± 0.48	12.42 ± 2.32 ^#^	6.76 ± 0.98 *

Values are means ± SE (each group, *n* = 10). ^#^ *p* < 0.05, ^##^ *p* < 0.01, ^###^ *p* < 0.001 versus ND group, and * *p* < 0.05, ** *p* < 0.01, *** *p* < 0.001 versus HFD group. HOMA-IR = serum insulin (μU/mL) × serum glucose (mmol/L)/22.5.

## Data Availability

The data presented in this study are available on request from the corresponding author.

## Supplementary Materials

The following supporting information can be downloaded at: https://www.mdpi.com/article/10.3390/nu15153457/s1, Table S1: Composition of experimental diet; Table S2: Kits used for analysis of biochemical parameters in serum; Table S3: Primer for qRT-PCR.
