# Supplementary material for: 6-Gingerol Ameliorates Adiposity and Inflammation in Adipose Tissue in High Fat Diet-Induced Obese Mice: Association with Regulating of Adipokines
Source: Nutrients. 2023 Aug 4;15(15):3457. doi: 10.3390/nu15153457 (PMC10421254; doi:10.3390/nu15153457)
Supplement: Supplementary file 1 [file nutrients-15-03457-s001.zip › nutrients-2534968-supplementary.pdf]

Supplementary Tables

Table S1. Composition of experimental diet (units: g/kg diet)

	ND	HFD	HFD+6G
Casein	200	200	200
Corn oil	50	50	50
Lard	-	50	50
Cocoa butter	-	70	70
Coconut oil	-	30	30
Cholesterol	-	5	5
Corn starch	350	195	195
Sucrose	300	300	300
Cellulose	50	50	50
Mineral mixture[1)]	35	35	35
Vitamin mixture[2)]	10	10	10
Methionine	3	3	3
Choline bitartrate	2	2	2
6-Gingerol	-	-	0.5

1) AIN-76 diet based mineral mixture
2) AIN-76 diet based vitamin mixture

Table S2. Kits used for analysis of biochemical parameters in serum

Biochemical parameter	Code	Manufacturer	
Triglyceride	1120261	Shinyang Diagnostics	Seoul, South Korea
Free fatty acids	MAK044	Sigma-Aldrich	St. Louis, Mo, USA
Total cholesterol	1120181	Shinyang Diagnostics	Seoul, South Korea
HDL-cholesterol	MAK045	Sigma-Aldrich	St. Louis, Mo, USA
Glucose	1120201	Shinyang Diagnostics	Seoul, South Korea
Insulin	EZRMI	Sigma-Aldrich	St. Louis, Mo, USA
Leptin	MOB00B	R&D Systems	Minneapolis, MN, USA
Adiponectin	MRP300	R&D Systems	Minneapolis, MN, USA

Table S3. Primer for qRT-PCR

Target gene	Primers	Sequence (5'→ 3')
PPARγ	F	GTCACGGAACACGTGCAGC
	R	CAGGAGCGGGTGAAGACTCA
C/EBPα	F	CAAGAACAGCAACGAGTACCG
	R	GTCACTGGTCAACTCCAGCAC
SREBP-1	F	ATCGCAAACAAGCTGACCTG
	R	AGATCCAGGTTTGAGGTGGG
FAS	F	ATCCTGGAACGAGAACACGATCT
	R	AGAGACGTGTCACTCCTGGACTT
CD36	F	GATGACGTGGCAAAGAACAG
	R	AAAGGAGGCTGCGTCTGTG
Adiponectin	F	GGAGAGAAAGGAGATGCAGGT
	R	CTTTCCTGCCAGGGGTTC
Leptin	F	CAGGATCAATGACATTTCACACA
	R	GCTCCTGAGGACCTGTTGAT
Resistin	F	TGCCAGTGTGCAAGGATAGA
	R	TGGAAACCACGCTCACTTC
TNFα	F	CCCTCACACTCAGATCATCTTCT
	R	GCTACGACGTGGGCTACAG
IL-6	F	TAGTCCTTCCTACCCCAATTTCC
	R	TTGGTCCTTAGCCACTCCTTC
MCP-1	F	CCACTCACCTGCTGCTACTCA
	R	TGGTGATCCTCTTGTAGCTCTCC
F4/80	F	CCCCAGTGTCCTTACAGAGTG
	R	GTGCCCAGAGTGGATGTCT
PAI-1	F	AGGATCGAGGTAAACGAGAGC
	R	GCGGGCTGAGATGACAAA
β-actin	F	AATACCCCAGCCATGTGTGT
	R	ATGGGCACTGTGTGTGACC
